# Enriching the international clinical nomenclature with Chinese daily used synonyms and concept recognition in physician notes

**DOI:** 10.1186/s12911-017-0455-z

**Published:** 2017-05-02

**Authors:** Rui Zhang, Jialin Liu, Yong Huang, Miye Wang, Qingke Shi, Jun Chen, Zhi Zeng

**Affiliations:** 1 Department of Medical Informatics, West China School of Medicine/West China Hospital, Sichuan University, Chengdu, Sichuan People’s Republic of China; 2 Information Center, West China Hospital, Sichuan University, Chengdu, Sichuan People’s Republic of China; 3 Department of Otorhinolaryngology, West China Hospital, Sichuan University, Chengdu, Sichuan People’s Republic of China; 4 Department of Ophthalmology, West China Hospital, Sichuan University, Chengdu, Sichuan People’s Republic of China; 5 Department of Cardiology, West China Hospital, Sichuan University, Chengdu, Sichuan People’s Republic of China

**Keywords:** Chinese term of clinical finding, SNOMED CT, Terminology localization, Synonyms enrichment, Clinical term recognition, Concept mapping of terminology

## Abstract

**Background:**

It has been shown that the entities in everyday clinical text are often expressed in a way that varies from how they are expressed in the nomenclature. Owing to lots of synonyms, abbreviations, medical jargons or even misspellings in the daily used physician notes in clinical information system (CIS), the terminology without enough synonyms may not be adequately suitable for the task of Chinese clinical term recognition.

**Methods:**

This paper demonstrates a validated system to retrieve the Chinese term of clinical finding (CTCF) from CIS and map them to the corresponding concepts of international clinical nomenclature, such as SNOMED CT. The system focuses on the SNOMED CT with Chinese synonyms enrichment (SCCSE). The literal similarity and the diagnosis-related similarity metrics were used for concept mapping. Two CTCF recognition methods, the rule- and terminology-based approach (RTBA) and the conditional random field machine learner (CRF), were adopted to identify the concepts in physician notes. The system was validated against the history of present illness annotated by clinical experts. The RTBA and CRF could be combined to predict new CTCFs besides SCCSE persistently.

**Results:**

Around 59,000 CTCF candidates were accepted as valid and 39,000 of them occurred at least once in the history of present illness. 3,729 of them were accordant with the description in referenced Chinese clinical nomenclature, which could cross map to other international nomenclature such as SNOMED CT. With the hybrid similarity metrics, another 7,454 valid CTCFs (synonyms) were succeeded in concept mapping. For CTCF recognition in physician notes, a series of experiments were performed to find out the best CRF feature set, which gained an F-score of 0.887. The RTBA achieved a better F-score of 0.919 by the CTCF dictionary created in this research.

**Conclusions:**

This research demonstrated that it is feasible to help the SNOMED CT with Chinese synonyms enrichment based on physician notes in CIS. With continuous maintenance of SCCSE, the CTCFs could be precisely retrieved from free text, and the CTCFs arranged in semantic hierarchy of SNOMED CT could greatly improve the meaningful use of electronic health record in China. The methodology is also useful for clinical synonyms enrichment in other languages.

## Background

A considerable amount of meaningful information is routinely recorded in an unstructured text format in clinical information system (CIS). Almost 50% of the medical records are physician free text notes [1]. The lack of standardization in how medical information coded is a major difficulty which hindering the application of clinical data [2]. SNOMED CT (Systematized Nomenclature of Medicine, Clinical Terms) is a systematically organized standard set of clinical terminology covering all areas of clinical information [3]. It helps the natural language processing (NLP) task in clinical concept recognition in some western languages. However, it has been shown that the entities in everyday clinical text are expressed in a way that varies from how they are expressed in the SNOMED CT (SCT) terminology [4]. The natural language of physician note does not follow the precise expressions in terminologies, and is typically written with abbreviations, misspellings or medical jargons [4]. Over a period of 40 years, fewer studies are available on the usage of SNOMED in clinical practice [5]. A terminology without enough synonyms may not be adequately suitable for the task of daily used clinical term recognition.

The attempt to change the habit of physician documentation from free-text to formatted computer interface has failed due to time-consuming and inconvenience of description [6]. Meanwhile, it is an urgent need for the physicians to retrieve text information for the purpose of clinical research [7]. For this reason, the work of retrieving synonyms and categorizing them into the standardized clinical terminology is a significant challenge all over the world. Most of the existing terminologies and document collections are in western language (mainly English), and the multilingual resources are far from being complete [8]. The task is more difficult for Chinese because of the language difference, such as the absence of nature spaces among Chinese words. The lacking of effective Chinese clinical terminology has become a major bottleneck for text information utilization in China [9]. Fortunately, once a clinical term in native language mapped to the SCT concepts, it could cross map to other international standards and classifications [10], such as the Unified Medical Language System (UMLS) and the Medical Subject Headings (MeSH). Nowadays, various studies have investigated for automatic methods to assist the translation of biomedical terminologies or create multilingual biomedical vocabularies [11]. Most studies however are focus on inter-terminology concept mapping based on a same language [12]. For the similarity in different European languages, the medical terminologies could be translated through word alignment in parallel text corpora [13], or translation algorithm based on affixes [14]. String matching against SCT terms was applied in countries which native language is similar to English [15–17]. However, most methods used to translate biomedical terms were rule-based and hand-coded, which were difficult in transferring to other languages or domains [8]. Unfortunately, mapping the Chinese terminology to English concept represents a greater difference from other western languages to English owing to different language features.

In 1990s, more than 70 Chinese scholars spent 10 years on translation the SNOMED III (also known as SNOMED International) from English to Chinese. The work involved was tremendous and did not update anymore. As a result, its electronic version is seldom used. Another project of SCT localization, which involves machine translation, manual review and artificially synonyms adding, is still under way in China [10]. Other than that, the Chinese Unified Medical Language System (CUMLS) [18] or the Unified Traditional Chinese Medical Language System (UTCMLS) [19] are far from been established. Be that as it may, the localization based on translation mainly includes precise expressions rather than daily used synonyms. With personal habit, a same concept expression may vary either in word sequence or abbreviation in Chinese. An ideal clinical term recognition system could depend on an over-complete nomenclature which contains enough synonyms collected from real-world clinical text. Instead of starting from scratch, we refer to the Chinese SNOMED III and Chinese ICD-10 (International Classification of Diseases 10th Revision), two referenced Chinese clinical nomenclatures which could cross map to other international nomenclatures, and intend to enrich them with daily used synonyms from CIS.

The methods of clinical concept recognition generally fall into three categories: dictionary-based methods, rule-based methods and statistical machine learning methods [20]. Dictionary-based methods search through medical nomenclature to recognize the information. They were apt to work but often suffer from low recall for absence of an over-complete nomenclature. Rule-based methods require experts to list hand-coded rules, regular expressions and exceptions. They were not easy to achieve because of time-consuming in corpus observation and rule refinement. The statistical machine learning methods, including the Hidden Markov Model (HMM), Support Vector Machine (SVM), Maximum Entropy Markov Model (MEMM), and Conditional Random Fields (CRF), are reliant on annotated clinical corpora, which are often unavailable for the patient privacy and confidentiality requirements. Fortunately, the i2b2 had shared some annotated clinical data for the NLP task, which attracted variety of systems for medical concept extraction. In the competition, all but one of the best effective clinical concept extraction systems used CRF approach [21]. The CRF also has been proven to be the state-of-art model among these machine learning models [22]. Besides, the terminology-based recognition methods were popular if the dictionaries (such as SCT or UMLS) are easy to include [23, 24]. For lacking of effective Chinese clinical nomenclature, Wang [25] compared three models (the CRF, HMM and MEMM) for the task of symptom name recognition in traditional Chinese clinical records. It also indicated that the CRF outperforms the other two methods.

This study aims to address these issues of enriching the Chinese clinical nomenclature. The task mainly focus on the *Clinical Finding* hierarchy, a most important hierarchy of the 19 top level hierarchies in SCT, which represents the clinical observation, disease and disorder, assessment or judgment, and both normal and abnormal clinical state. Three aims of this research are:

- To retrieve the daily used Chinese term of clinical finding (CTCF) from physician notes in CIS.
- To map the CTCFs to the corresponding SCT concepts, which helps the SNOMED CT with Chinese synonyms enrichment (SCCSE).
- To recognize the CTCFs in Chinese physician notes in CIS.

## Methods

**Fig. 1:**
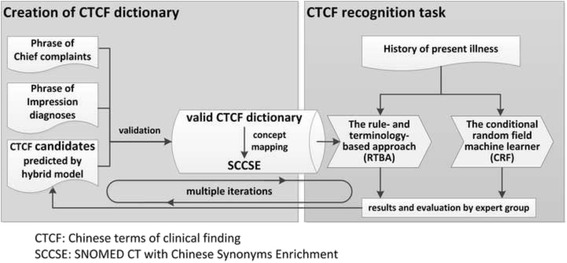
The system framework

The first task is to enrich the SCT with Chinese synonyms, which includes the CTCF retrieving, categorization, and concept mapping. The second task is the CTCF recognition in physician notes (Fig. 1).

### Enriching the SNOMED CT with Chinese synonyms

#### Initial CTCF dictionary creation

For the absence of nature space among Chinese words, the word segmentation in complex clinical sentences is a great risk. However, for initial CTCF dictionary creation, huge amounts of brief sentences describing CTCFs, such as Chinese chief complaint (CCC) and Chinese impression diagnosis (CID), could be first analyzed. After a rule-based algorithm on them, some phrases (or potential CTCFs) could be roughly retrieved, known as CTCF candidates. Since most of CTCF candidates did not meet the CTCF criteria, only a subset of them were accepted as valid CTCFs across manual validation. Afterwards, the complex sentences such as history of present illness (HPI) were analyzed after the initial CTCF dictionary created.

##### The CTCF candidates retrieving from the CCC

The CCC is a regular expression of main syndromes and signs that suffered by patients. The CCC usually owns a fixed format as ‘syndrome and its durations’, e.g. ‘咽痛、吞咽梗阻3 + 月’ (Feeling of sore throat, swallow obstruction for more than 3 months). A rule-based algorithm was designed to automatically retrieve the CTCF candidates. The key words groups were carried out in Table 1.

**Table 1:** Key words group for CTCF retrieving and recognition

Key words group	Function	Example
Time Period	Matching the time duration phrase.	年(year),月(month),周(week),天(day),小时(hour)…
Number	Matching the numbers in describing time duration.	1,2,3,4,5,6,7,8,9,0,半(half),一(one),二(two),三(three),+(more than)…
Modifier in CTCF	Some modifiers which mingled within CTCF could be ignored in the recognition task.	持续(constantly),逐渐(gradually),明显(obviously), 稍显(slightly),反复(recurrently)…
Exception with context	Some phrases, even matched, were invalid with context.	抗乙肝药物(anti-HBV drugs), 多尿期(the polyuria stage),最高血压(the highest blood pressure)…

The CCC was firstly segmented into clauses by punctuations, both in full-width form (Chinese format, a double byte character set, e.g.,。:;“”、) and half-width form (English format, a single byte character set, e.g.,.:;””). Afterwards, each character in the clause was reversely matched by the word group of *‘Time Period’* and *‘Number’* iteratively to recognize and remove the phrase of time duration. After that, only the CTCF candidates left.

##### The CTCF candidates retrieving from the CID

The CID for outpatients mainly includes diagnoses, disorders and syndromes. It consists of brief phrases instead of a whole sentence. Each case of CID was separated into more short phrases by any punctuation except the bracket and hyphen. All short phrases, which occurred more than 20 times in the 22.5 million cases of CID, were called CTCF candidates and waiting for manual review.

##### A novel approach for new CTCF candidate prediction

At the end stage of this research, two independent CTCF recognition approaches, the Rule- and Terminology-Based Approach (RTBA) and the Conditional Random Field (CRF) machine learner, were combined to discover new CTCFs in HPI. All CTCFs which marked by CRF but unmarked by RTBA, were artificially reviewed. About one-third of them were ultimately confirmed to be valid CTCFs. It indicated that it’s an efficient way to discover new CTCFs. After several iterations, more CTCF candidates could be located. The details would be introduced in the following section.

##### Manual validation of the CTCF candidates

After sorting these CTCF candidates by frequency of occurrence, the physicians reviewed and validated them in descending order. Each CTCF candidate was assessed independently by two physicians using the standardized evaluation criteria. A third physician reviewed by adjudication, in cases of disagreement. The CTCF evaluation criteria was: (a) Symptoms and signs; (b) Diseases and disorders; (c) All positive and negative findings from medical test, examination or laboratory; (d) Both normal and abnormal clinical states; (e) Clinical assessments or judgments; (f) Exclusion of the phrases which containing any punctuation.

#### CTCF categorization and concept mapping

To solve the problem of ‘one clinical concept with different descriptions’, SCT concepts are further described by various *Descriptions*, which do not have to be unique or unambiguous. In real would, lots of modifiers mingled within daily used CTCF. For example, in the CTCF ‘右耳剧烈疼痛’ (severe pain in right ear), the words ‘severe’ and ‘right’ are modifiers which may not exist in standard description. All CTCFs with different modifiers but mean the same concept should be assigned to a same category. Extraction of synonyms and concepts has been approached using a variety of methods [26]. In our research, we established the correspondence between two CTCFs based on hybrid similarity including literal similarity (LS) and diagnosis-related similarity (DS).

##### Literal similarity metrics

The literal similarity depends on Jaro Distance (JD), Jaro-Winkler Distance (JWD), and Adjust Jaro-Winkler Distance (AJWD). The JWD, a variant of the JD metric, uses a prefix scale *PreScale* which gives more favourable ratings to strings that match from the beginning for a set prefix length *preLen*. Given two strings S_1_ and S_2_, their Jaro-Winkler distance is:

1 $$ J W D\left({s}_1,{s}_2\right)= J D\left({s}_1,{s}_2\right)+ preLen\cdot preScale\cdot \left(1- JD\left({s}_1,{s}_2\right)\right) $$

The *preLen* is the length of common prefix at the start of the string. The *PreScale* is a constant scaling factor for how much the score is adjusted upwards for having common prefix. Classically, most syndromes consist of body part (prefix) and sensation (suffix). Unlike western language, the common suffix in two CTCFs often means a same sensation with different body parts, which is more likely to describe a similar clinical problem. So we redefined the *preLen* of JWD and adjusted it to the length of common suffix, as Adjust Jaro Winkler Distance (AJWD). The literal similarity was calculated by JWD or AJWD, whichever is higher.

##### Diagnosis-related similarity metrics

Each disease or diagnosis leads a fixed group of syndromes, signs, and disorders. On the contrary, a certain syndrome, sign, or disorder usually caused by some fixed disease. Based on plenty of medical records in CIS, the corresponding discharge diagnoses of two CTCFs are taking into account to measure whether they express a similar concept. In most hospitals, the discharge diagnosis is coded by ICD-10. Naturally, the Term Frequency-Inverse Document Frequency (TF-IDF) weighting scheme [27] is involved to measure the influences of a diagnosis to a particular CTCF. Each discharge diagnosis could be expressed by its three-character category (such as C20), four-character subcategory (such as C20.1), or even all-character ICD code. The more the characters considered, the more diversify the diagnosis group is. On account of all discharge diagnoses (not only the main discharge diagnosis) were taken into account, the three-character category was chosen to represent each disease group, avoiding the problem of data sparsity.

In TF-IDF schema, we let *t*_*i*_ be a CTCF, *D*_*i*_ be its corresponding discharge diagnoses group which containing all the occurrences of diagnoses in medical records with the CTCF *t*_*i*_. And *D* is the set of all diagnoses in the research. For any *d*_*j*_∈*D*, its weight *w*_*i,j*_ for *t*_*i*_ is calculated as:

2 $$ {w}_{i, j}= t{f}_{i, j}\times id{f}_j $$

3 $$ t{f}_{i, j}=\frac{n_{i, j}}{{\displaystyle \sum_k{n}_{k, j}}} $$

4 $$ id{f}_j=\frac{\left| D\right|}{d{f}_j} $$

The *n*_*i,j*_ is the number of occurrence of *d*_*j*_ in *D*_*i*_, |*D*| is the number of diagnoses in *D*, and *df*_*j*_ is the number of CTCF whose corresponding discharge diagnoses group contain *d*_*j*_. All CTCFs with negative expression won’t be involved in TF-IDF schema. After that, a vector in multi-dimensional space was constructed by the weighted diagnoses to describe every CTCF. For two CTCFs *t*_*m*_ and *t*_*n*_, if their corresponding diagnosis groups were *D*_*m*_ and *D*_*n*_, the following vectors were used to describe *D*_*m*_ and *D*_*n*_.

5 $$ {V}_m=\left\langle {w}_{m,1},{w}_{m,2},\dots, {w}_{m,\left| D\right|}\right\rangle $$

6 $$ {V}_n=\left\langle {w}_{n,1},{w}_{n,2},\dots, {w}_{n,\left| D\right|}\right\rangle $$

Finally, the diagnosis-related similarity for CTCF *t*_*m*_ and *t*_*n*_ was calculated by the cosine metric.

7 $$ S i{m}_{Diag- rela}\left({c}_m,{c}_n\right)= \cos \left({V}_m,{V}_n\right)=\frac{{\displaystyle \sum_{k=1}^{\left|\mathrm{D}\right|}{w}_{m k}\cdotp {w}_{n k}}}{\sqrt{{\displaystyle \sum_{i=1}^{\left|\mathrm{D}\right|}{w}_{m i}^2}}\cdotp \sqrt{{\displaystyle \sum_{j=1}^{\left|\mathrm{D}\right|}{w}_{n j}^2}}} $$

##### Hybrid similarity metrics

The Hybrid Similarity (HS) metrics of two CTCFs is calculated as quadratic sum (QS) or arithmetic sum (AS) separately.

8 $$ S i{m}_{QS}\left({C}_m,{C}_n\right)=\sqrt{\beta \cdotp S i{m}_{Literal}^2\left({C}_m,{C}_n\right)+\left(1-\beta \right) Si{m}_{Diag- rela}^2\left({C}_m,{C}_n\right)} $$

9 $$ S i{m}_{AS}\left({C}_m,{C}_n\right)=\beta \cdotp S i{m}_{Literal}\left({C}_m,{C}_n\right)+\left(1-\beta \right) S i{m}_{Diag- rela}\left({C}_m,{C}_n\right) $$

The *β* was firstly set to 0.6 for empirical observation. Only a pair of CTCFs, whose hybrid similarity above a certain threshold, was considered ‘synonym pair’ candidates and waited for manual review.

##### Concept mapping of CTCF

A referenced CTCF (rCTCF) means a CTCF which is accordant with the description in referenced Chinese clinical nomenclature (rCCN), which could cross map to other international nomenclature such as SCT. Two existing rCCNs were eligible to serve as the resources in this research.

- The Chinese SNOMED III. It contains 145,856 descriptions, each of which owns a concept ID to SCT. The descriptions in hierarchy of F (function) and D (Diseases/Diagnoses) were considered the rCTCFs, totally 44,862 descriptions.
- The Chinese ICD-10. It contains 28,668 descriptions, which could cross map to SCT. Besides the classification of disease, the Chapter R contains symptoms, signs and abnormal clinical and laboratory findings. All descriptions in Chinese ICD-10 were considered the rCTCFs.

These rCTCFs in rCCNs could help the aim of SNOMED CT with Chinese synonyms enrichment (SCCSE). All CTCFs were calculated the hybrid similarity with each rCTCF. After that, each CTCF was provided a candidate queue, in which the rCTCF candidates listed in descending order by HS score. The rCTCF candidate queue allowed the experts to quickly proceed through. The experts could either approve any, or reject all system-mappings and artificially adjust them. Afterwards, with approved mapping results, the CTCFs could map to the corresponding SCT concepts via rCTCF.

### CTCF recognition task in clinical text

Two methods for CTCF recognition were applied in physician notes.

#### The rule- and terminology-based approach for CTCF recognition

Physicians are trained to use the normalized clinical term and refine the language. A terminology-based recognition method was conceivable if an integrated lexicon is available. With a CTCF dictionary, the Chinese clinical term could be retrieved with high precision through exact string matching. A rule- and terminology-based approach (RTBA) was designed, and the reverse maximum matching (RMM) and several types of key word list (Table 1) were carried out in RTBA to increase the matching accuracy. The following steps were involved in RTBA.

- Each sentence in HPI was segmented into short clauses by all types of punctuation, except the caesura sign. For each short clause, the RMM was used to recognize the CTCF. Considering the longest CTCF containing 16 characters, a same character context window was adopted.
- Some CTCFs, even matched by RMM, were invalid with context. For example, the CTCF ‘多尿’ (polyuria) is a substring of another phrase of ‘多尿期’ (the polyuria stage), but should not be recognized as a valid CTCF in this context. A hand-built key word group of *‘Exception with context’* (Table 1) was created to solve this problem.
- To refine the language, some aggregated CTCFs are created. The aggregated CTCF may include several independent CTCFs which shared a same prefix or suffix. For language refined, the redundant parts, either prefix or suffix, would appear once for short. For example, the phrase ‘颈肩部麻木’ (the neck and shoulder are numb) is a refined aggregated CTCF which actually consists of two individual CTCFs ‘颈部麻木’ (the neck is numb) and ‘肩部麻木’ (the shoulder is numb). A sub procedure in RTBA worked for detecting these aggregations. Once a CTCF matched by RMM, a sub procedure would scan and locate other potential prefix or suffix which may exist in the neighboring characters. The sub procedure was:
  - In stage of CTCF dictionary creation, each CTCF owned a corresponding group of ‘potential prefix’ and ‘potential suffix’. For example, if two CTCFs had a same prefix (or suffix), the rest part of them would add into the group of potential suffix (or prefix) of each other.
  - Once a CTCF matched by RMM, each potential prefix (or suffix) of the CTCF would try to match the neighboring characters. If success, the frequency of occurrence of potential prefix (or suffix) increased 1.
  - Finally, the frequently occurred potential prefixes (or suffixes) with each particular CTCF were reviewed by experts, judging whether the aggregations were accepted.
- The judgment whether a CTCF expressing a negative meaning is a separate experiment. The CTCFs with negative meaning are mostly indicated by a limited group of Chinese key words such as ‘不伴,无,否认,未,消失,不明显’, which mean ‘without’.

Finally, the outcome of the RTBA was organized in a BIEO format. Each character was labeled with a B (beginning of a CTCF), I (inside a CTCF), E (end of a CTCF) or O (not member of a CTCF).

#### Conditional random rields for CTCF recognition

A CRF machine learner was used for CTCF recognition. For the absence of nature spaces between Chinese words, the characters are viable alternatives to words as the features to avoid the inappropriate word segmentation. We know that the tokenization and part of speech (POS) tagging developed for general text may not be adequate for biomedical domain [26]. Yet, in order to get more word features which might be useful, ICTCLAS (Institute of Computing Technology, Chinese Lexical Analysis System) [28], the most famous lexical analysis analyzer for Chinese general text, was used to determine the word boundary and POS tag. The ICTCLAS is based on hierarchical hidden Markov model, with precision and recall over 90%.

In order to find out the best feature set to feed to the CRF machine learner, several features were involved to test the performance.

- Context feature: The characters around the target character are important for CRF utilization. The neighboring characters may provide useful information and help in predicting the correct tag for each token. The context window size (CWS) means in addition to the character itself, how many characters around it are used as feature for output tags predicting.
- N-gram feature: Chinese character unigram (U), bigram (B), and trigram (T) cover most of the meaningful words in clinical text. Considering lots of long fixed expressions in Chinese, the quadgram (Q) was also taken into account.
- Stop character feature: Some characters which frequently occurred in clinical text would never appear in any valid CTCF.
- Grammatical feature: The ICTCLAS was used for tokenization and producing the grammatical features. The POS tags for each word may assist in determining the CTCF boundaries.
- Associative strength feature: It common practice to classify words on their co-occurrence with other words [26]. We use the chi-square statistic to test the association between two adjacent words based on the 218-million characters clinical corpus. In this research, the significance level is 0.05 and the critical value is 3.841. Two adjacent words were considered combined only if *X* ^*2*^ > 3.841.

In CRF training data, each column represents a feature and the last column represents the output tag which annotated by physicians. In order to find out the best feature set, each feature was individually added into the baseline model (only context feature contained) to calculate the F-score increasing. After sorting these features by descending order, the ‘best’ features were sequentially added. Any feature increasing the F-score would be retained, otherwise it was removed.

### Data and experimental settings

The material for this research consists of de-identified physician notes from CIS, which includes:

- 520 thousand cases of CCC and HPI with discharge diagnoses from 2011.1 to 2014.10
- 22.5 million cases of CID for outpatient from 2008.3 to 2014.10.

Firstly, the three annotators jointly 1) annotated 20 cases of HPI, 2) validated 800 CTCF candidates, and 3) mapped 200 valid CTCFs to rCTCFs, to produce initial CTCF annotation and evaluation criteria. The criteria was then refined after several iterations. At the end of each iteration, all disagreements were discussed and the criteria would be revised if necessary. Finally, the evaluation agreements reached a stable state for each annotator. Then the CTCF annotation work was carried out on randomly chosen 1,000 cases (415,557 tokens) of HPI. Only a CTCF accepted by first two annotators (physician JC and RZ) independently would be accepted as valid, a third annotator (physician JL) reviewed the inconsistent labels and made the adjudication. The CTCF candidates which 1) occurred more than 10 times in CCC, 2) more than 20 times in CID, 3) all from hybrid approach of RTBA and CRF, were manually validated. Besides, any CTCF with highest HS score > 0.3 was also manually reviewed for concept mapping with rCTCFs.

The agreement of the first two annotators on validation and mapping task was measured by Kappa, which achieved 0.92 and 0.87 respectively. Since Kappa requires the number of false negatives, it cannot be used to measure agreement on annotation task which lacks well-defined negative cases. Therefore, the agreement of the first two annotators on annotation task was measured in terms of F-score, which reached 0.81.

A significant challenge for clinical ontology enrichment is the absence of systematic evaluation methods and reference standards [26]. For lacking of gold standard to estimate whether a CTCF is appropriately mapped to the corresponding concept on the ontology level, we only: 1) present the general picture of the CTCFs retrieved, and 2) evaluate the similarity metrics against manual annotation.

Besides, we evaluated the result of CTCF recognition with precision (P), recall (R), and F-score. These metrics rely on true positive (TP), false positive (FP), and false negative (FN).

10 $$ Precision=\frac{TP}{TP+ FP} $$

11 $$ Recall=\frac{TP}{TP+ FN} $$

12 $$ F\hbox{-} \mathrm{score}=\frac{2\times Precision\times Recall}{Precision+ Recall} $$

The research adopted the CRF++ tool with default settings [29]. Five hundred cases of annotated HPI (215,780 tokens) were used as the training set, and the other 500 cases (199,777 tokens) as the test set.

## Results

### General picture of CTCF retrieved and concept mapping

**Fig. 2:**
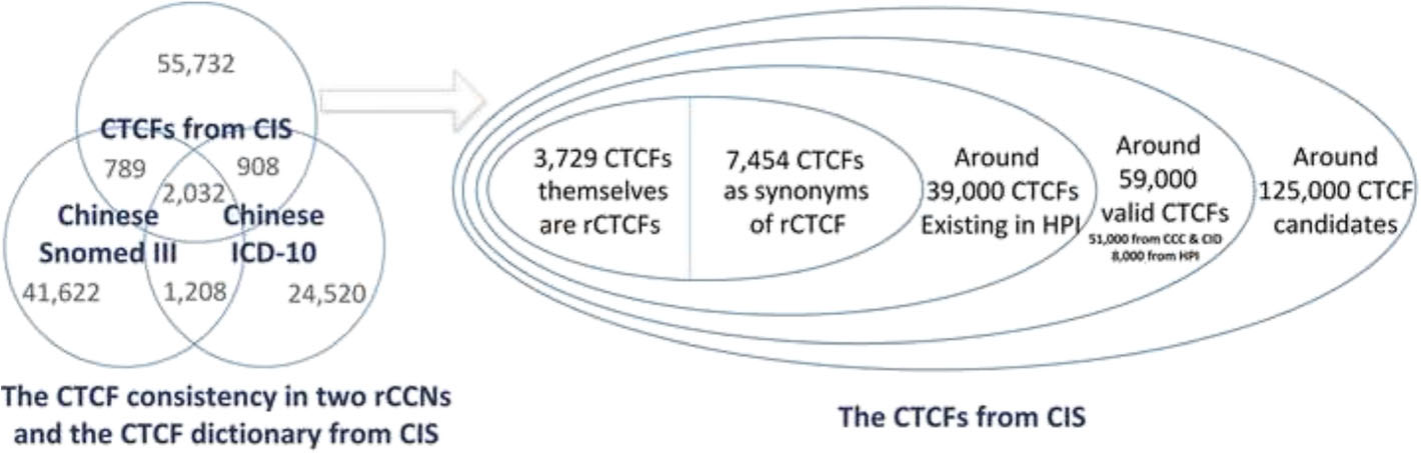
General picture of rCTCF and CTCF retrieved

**Fig. 3:**
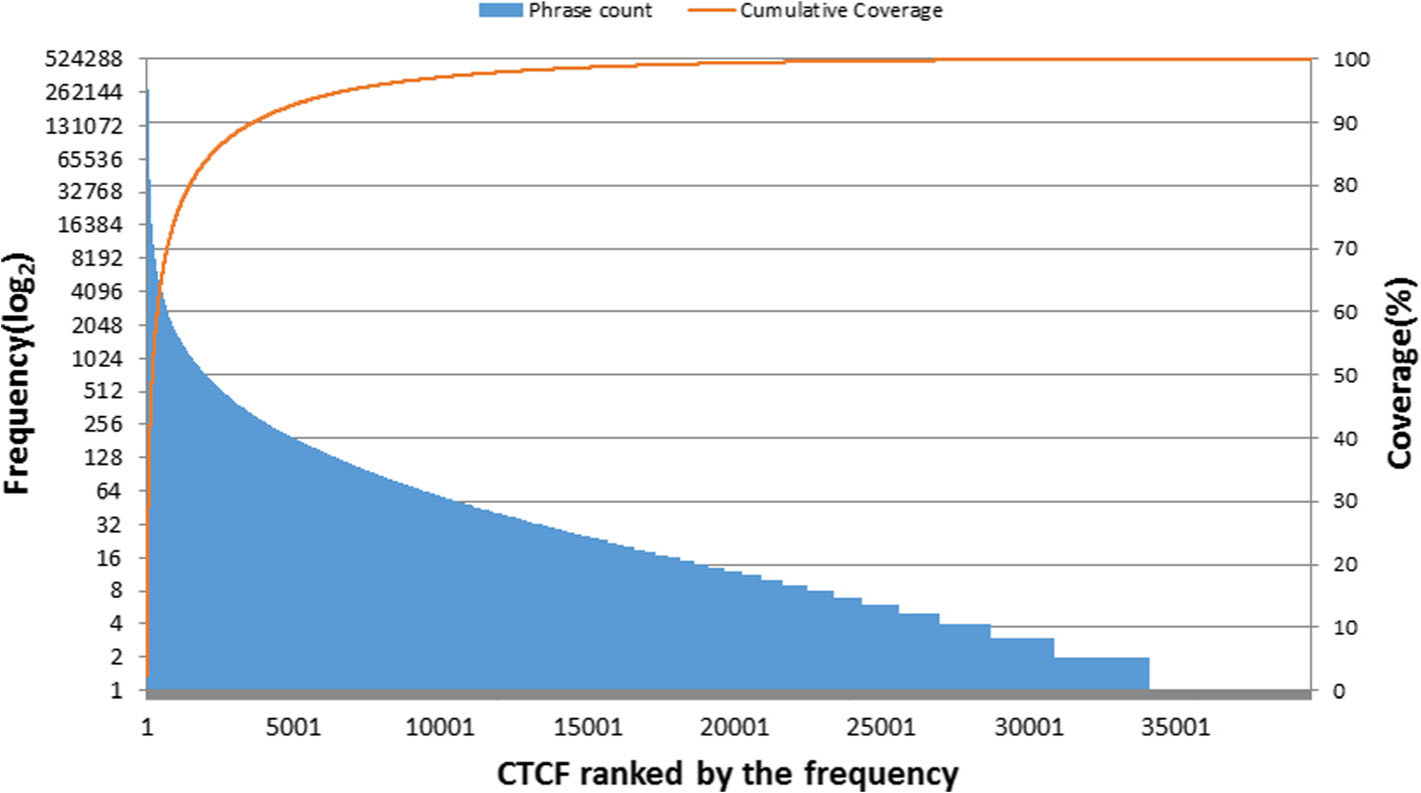
CTCFs sorted by frequency of occurrence in HPI

**Fig. 4:**
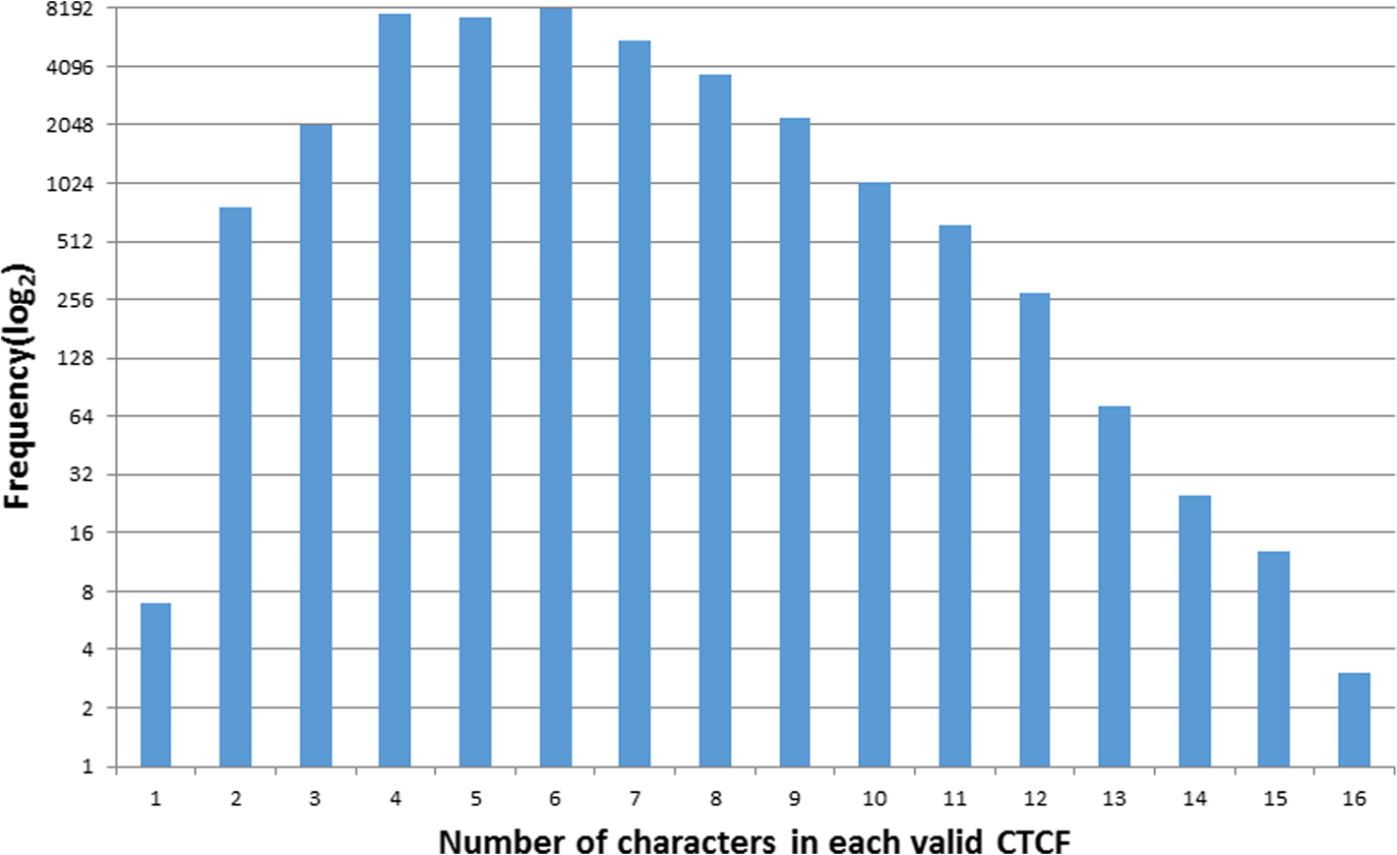
Number of characters in each valid CTCF in HPI

Since CCC and CID are both brief sentences, it is a straightforward process to collect the CTCF candidates. With human acting as a gatekeeper in CTCF validation (most CTCF candidates were manually reviewed), the accuracy of CTCF candidates retrieved was not evaluated. To sum up, there were almost 125,000 of CTCF candidates collected from the retrieving task of CCCs and CIDs. After manual validation, 51,000 of them were accepted as valid, as the initial CTCF dictionary. From the HPI, the hybrid method which combined the RTBA and CRF was applied to predict new CTCFs besides initial CTCF dictionary. With the hybrid method, another 8,000 CTCFs, which only occurred in HPI, were collected as a supplementary. As a result, total 59,000 valid CTCF were accepted (Fig. 2). To investigate their frequency of occurrence in HPI, another preliminary test was carried out and almost 39,000 of them occurred at least once in 520 thousand cases of HPI. As illustrated in Fig. 3, the horizontal axis represents the CTCF sorted from 1 to 39,000 by their frequencies. The bar graphs show each CTCF frequency in log_2_ scale (left vertical axis) and the line graph shows the cumulative percentage of phrase frequency over all CTCF occurrence (right vertical axis). The most frequent CTCF is ‘发热’ (fever), which annotated 280,964 times and constitutes 2.31% of all CTCF occurrences. The top 6,818 CTCFs cover 95% of all CTCF occurrences, which indicates that the CTCFs are characterized by skewness distribution. As shown in Fig. 4, more than 82% of the valid CTCFs consist of 4–8 Chinese characters with the median length at 6 characters, and the longest CTCF contains 16 characters.

The concept mapping was a time-consuming task. Only 3,729 valid CTCFs were accordant with the description in rCCN (Fig. 2). It demonstrated again that the entities in everyday clinical text are often expressed in a way that varies from the nomenclature. It is a surprise that only 3,240 out of 71,079 descriptions were consistent in two rCCNs. It reveals that the Chinese synonyms for clinical finding are still varies widely from rCCN to rCCN. This may be caused by the fact that Chinese ICD-10 is more a classification system than a nomenclature system, and Chinese is character-based language rather than word-based of western languages.

After manually reviewed, 7,454 CTCFs (as synonyms) have been mapped to the 1,683 rCTCFs. At closer observation, there are four relationships between CTCF and rCTCF:

- One-to-one: It’s the most common relationship.
- One-to-many: Some aggregated CTCFs like ‘主动脉瓣双病变’ (Double lesions of aortic valves) should be mapped to more than one concept such as ‘aortic stenosis’ and ‘aortic insufficiency’.
- Many-to-one: The average count of synonyms for each mapped rCTCF is 4.4.
- One-to-zero: As mentioned above, there were still 70% CTCFs that failed in mapping to any rCTCF. The reasons were: 1) there was a limitation of rCCNs coverage. 2) Both rCCNs were translational version, with formal, precise and academic expression, comparing with the more complex daily expression in real world. 3) Some CTCFs from medical imaging contained detailed anatomic sites, which difficult in concept mapping, such as ‘股骨髁上髁间开放粉碎骨折’ (open comminuted fracture of supracondylar and intercondylar femur). The introduction of Body Structure, another top hierarchy of SCT, might be helpful. 4) The similarity metrics are still worth further research, for some synonyms might be missed by low HS score.

Table 2 shows the system-mapping results of rCTCF candidates for each CTCF. Though some rCTCF candidates have a high HS score, they may not be synonyms. The reason is that the more general category (three-character category of ICD diagnoses) is liable to cause a high score in DS, which increases the false positive in synonym detection. The more detailed category (four-character subcategory) would help in discrimination, but this brings computational complexity and data sparsity.

**Table 2:** Some system-mapping results of rCTCF candidates for each CTCF

CTCF	The 1st rCTCF candidate	The 2nd rCTCF candidate	The 3rd rCTCF candidate	The 4th rCTCF candidate	…
主动脉夹层 (Aortic dissection)	主动脉夹层动脉瘤:0.91 (Aortic dissection aneurysm)	主动脉扩张:0.75 (Aortic dilation)	腹主动脉动脉瘤:0.75 (Abdominal aorta aneurysm)	主动脉瓣关闭不全:0.67 (Aortic valve insufficiency)	…
共同性外斜 (Concomitant exotropia)	共同性外斜视:0.95 (Concomitant exotropia)	共同性内斜视:0.89 (Concomitant esotropia)	共同性斜视:0.81 (Concomitant strabismus)	间歇性外斜视:0.70 (Intermittent exotropia)	…

The Hybrid Similarity (HS) score was followed with each rCTCF candidate

**Fig. 5:**
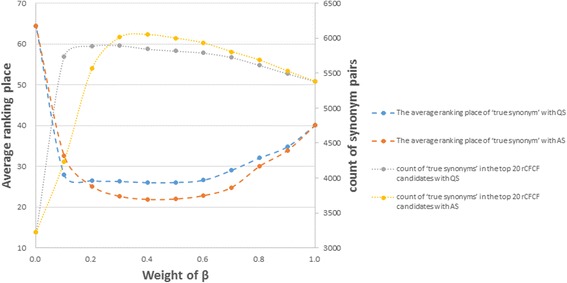
Average ranking place of ‘true synonym’ in the rCTCF candidates with different weights

To investigate the metrics proposed for concept mapping, two types of hybrid similarity metrics (AS and QS) are compared with different weights (the β from 0 to 1) to find out the best combination. Obviously, the higher ranking place of the ‘true synonym’ in the rCTCF candidates is important for manpower saving. From Fig. 5, with the hybrid similarity metrics by arithmetic sum and β = 0.4, the average ranking place is best (ranked 21.81 out of 3,729 rCTCF alternatives), and the top 20 rCTCF candidates also cover the most ‘true synonyms’ (6,052 out of 7,454). Obviously, the hybrid similarity metric is more appropriate than the literal similarity or diagnosis-related similarity alone.

**Fig. 6:**
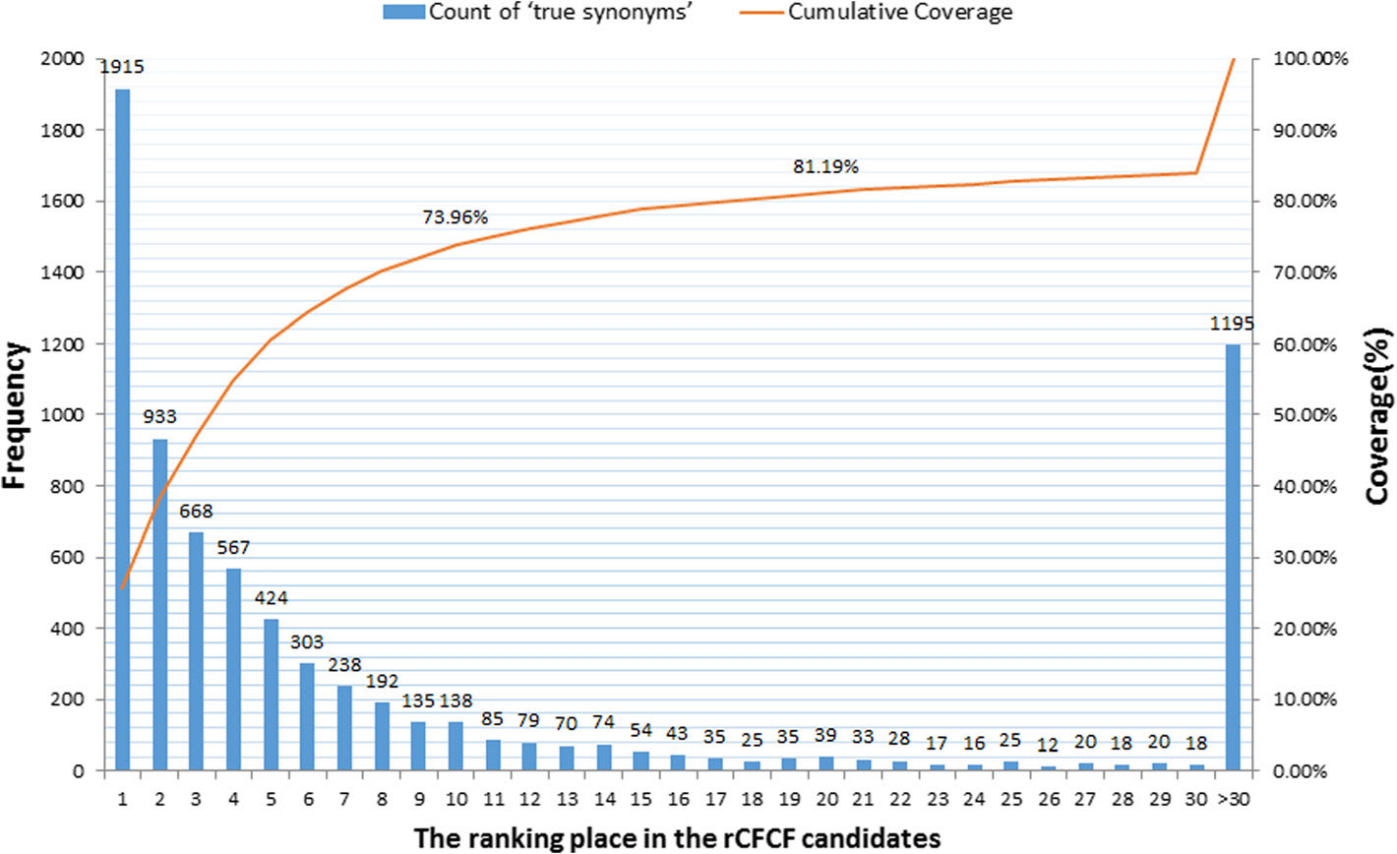
The ranking place of ‘true synonym’ in the rCTCF candidates

From Fig. 6, we also notice that 25.79% of ‘true synonyms’ could be located in the first rCTCF candidates, and 73.96% in the top 10 rCTCF candidates, and 81.19% in the top 20 rCTCF candidates. It indicated that merely review of the top 20 rCTCF candidates could locate most of ‘true synonyms’, which do save labor indeed.

**Fig. 7:**
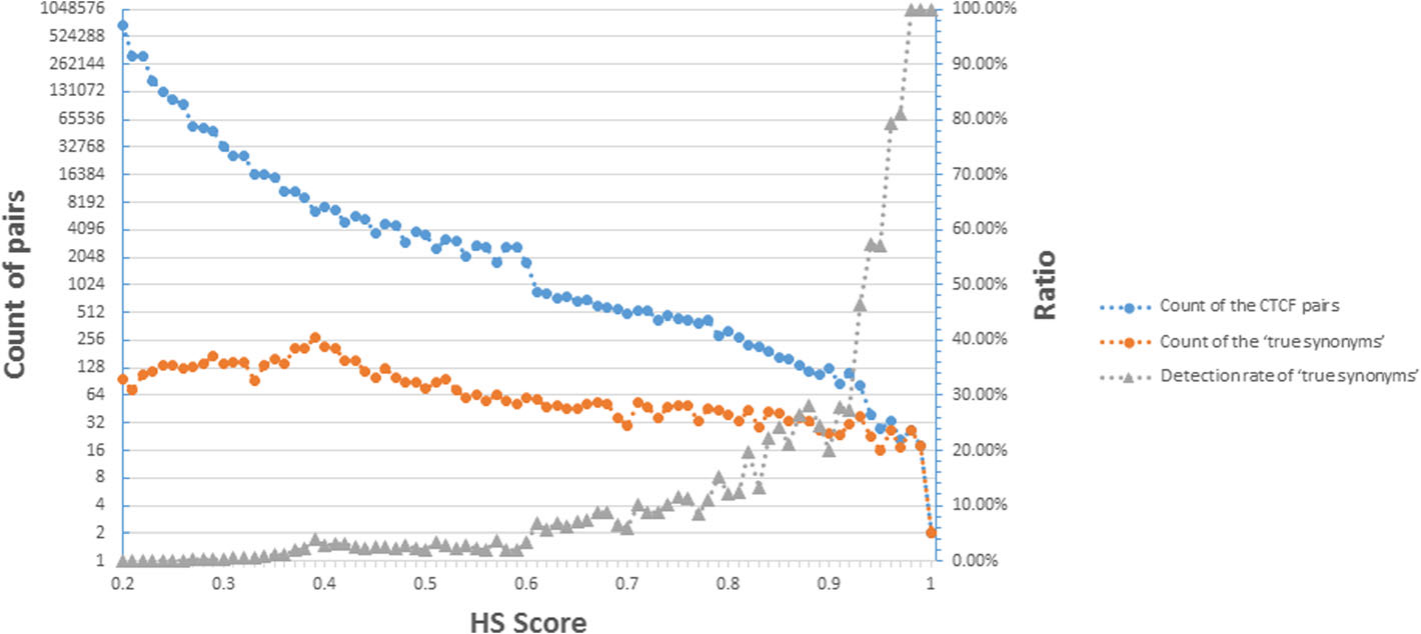
Detection rate of ‘true synonyms’ in each HS score

Figure 7 also demonstrates that there is a high detection rate of ‘true synonym’ (from 46.34% to 100%) when HS score > 0.94. With HS score decreased, the detection rate also shrinks. The detection rate decreases to 19.74% when HS score = 0.82, and 6.77% when HS score = 0.61. So, merely review of the synonym pairs whose HS > 0.61 could let the limited human labor play a biggest role.

### Evaluation of CRF for CTCF recognition

**Fig. 8:**
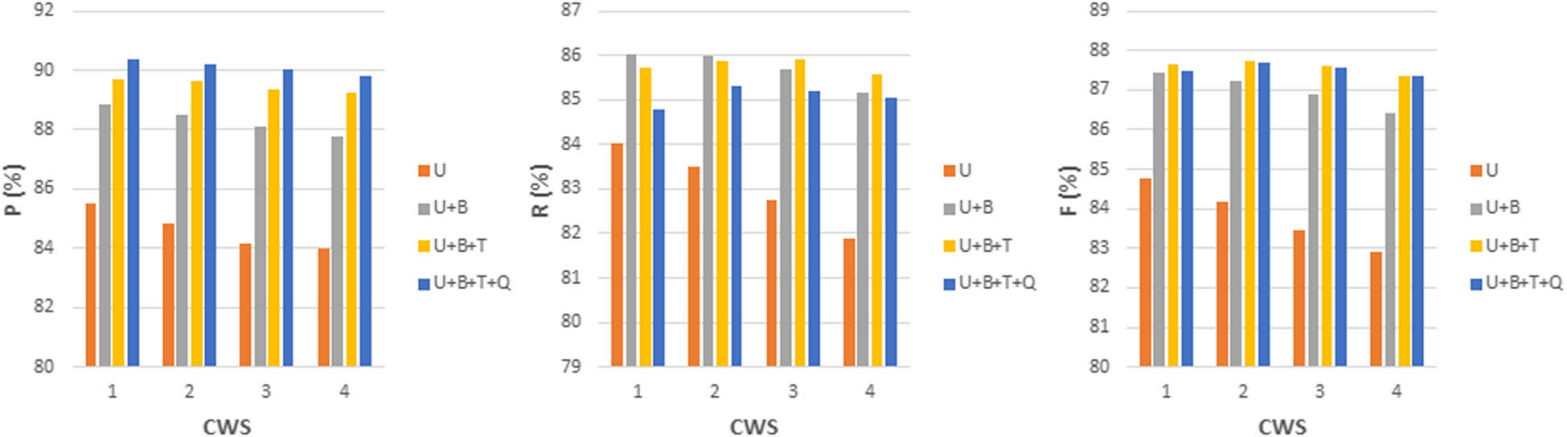
The performance of CRF with feature CWS and n-gram

Different feature sets in each conceivable combination have been tested to find out the best CRF model. If just considering the character features of CWS and n-gram, the best F-Score reached 0.877 and the similar performance (F-Score > 0.875) also achieved when n-gram = UBT or UBTQ and CWS = 1, 2 or 3, as shown in Table 3 and Fig. 8. That indicates when Chinese character is the only alternate for CRF feature, the CWS plays a limited role in CTCF recognition task. With the n-gram feature added, the performances of P, R, and F-score are all greatly improved, especially the trigram and quadgram.

**Table 3:** The performance of CRF with feature CWS and n-gram

Models	TP	FP	FN	P	R	F	Total
CWS1 + U	9427	1598	1791	0.855	0.840	0.848	11,218
CWS1 + UB	9650	1210	1568	0.889	0.860	0.874	11,218
CWS1 + UBT	9616	1104	1602	0.897	0.857	0.877	11,218
CWS1 + UBTQ	9510	1012	1708	0.903	0.848	0.875	11,218
CWS2 + U	9367	1676	1851	0.848	0.835	0.842	11,218
CWS2 + UB	9646	1250	1572	0.885	0.860	0.872	11,218
CWS2 + UBT	9635	1111	1583	0.897	0.859	0.877	11,218
CWS2 + UBTQ	9571	1042	1647	0.902	0.853	0.877	11,218
CWS3 + U	9282	1745	1936	0.842	0.827	0.835	11,218
CWS3 + UB	9614	1296	1604	0.881	0.857	0.869	11,218
CWS3 + UBT	9637	1145	1581	0.894	0.859	0.876	11,218
CWS3 + UBTQ	9559	1057	1659	0.900	0.852	0.876	11,218
CWS4 + U	9184	1749	2034	0.840	0.819	0.829	11,218
CWS4 + UB	9553	1331	1665	0.878	0.852	0. 864	11,218
CWS4 + UBT	9599	1156	1619	0.893	0.856	0.874	11,218
CWS4 + UBTQ	9542	1083	1676	0.898	0.851	0.874	11,218

**Fig. 9:**
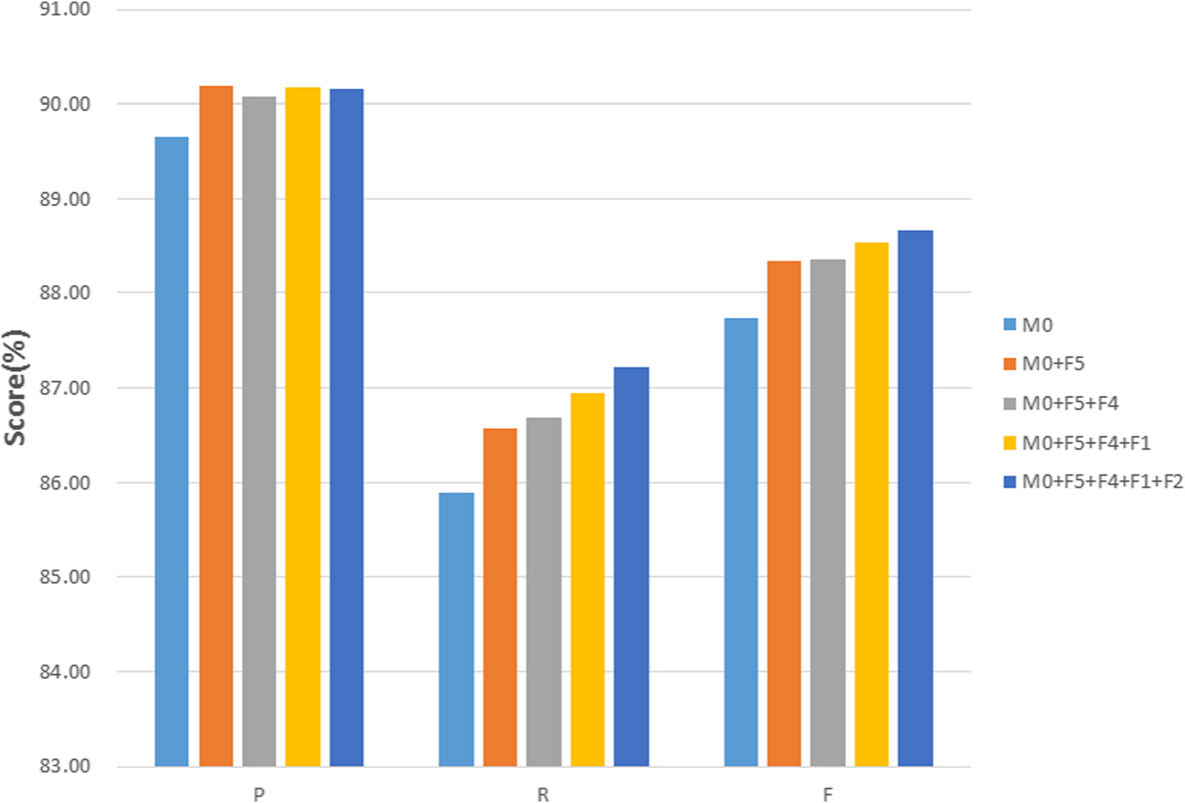
The performance of CRF with all features for CTCF recognition. F1: the stop character feature, F2: the current word feature, F3: the current and context word feature, F4: the word POS tag feature, F5: the word associative feature

Besides the character features, a series of word expanded features were also evaluated. With more word features added, the CRF model was elaborated and the results were improved. As shown in Table 4 and Fig. 9, the CRF model (with n-gram = UBT and CWS = 2) was chosen as the baseline model (M0). Firstly, when the associative strength feature added (M1), it not only improved the F-Score from 0.877 to 0.883, but also improved P and R. In model M2, the POS tag of the current word was added and the F-Score gained a slightly improvement. Afterwards, the feature of stop character was added in model M3 and leaded another 0.19% improvement of F-Score. Finally the current word feature was added (M4), and the best performance was recorded at the F-score of 0.887. The current and context word feature (F3) didn’t improve the last round performance and thus was removed from the best feature set.

**Table 4:** The performance of CRF with all features for CTCF recognition

Round	Models	TP	FP	FN	P	R	F	Total
-	M0 (baseline)	9635	1111	1583	0.897	0.859	0.877	11,218
1st	M0 + F1	9654	1135	1564	0.895	0.861	0.877	11,218
1st	M0 + F2	9671	1127	1547	0.896	0.862	0.879	11,218
1st	M0 + F3	9664	1135	1554	0.895	0.862	0.878	11,218
1st	M0 + F4	9678	1101	1540	0.898	0.863	0.880	11,218
1st	M0 + F5 (M1)	9711	1057	1507	0.902	0.866	0.883	11,218
2nd	M1 + F1	9717	1066	1501	0.901	0.866	0.883	11,218
2nd	M1 + F2	9725	1083	1493	0.900	0.867	0.883	11,218
2nd	M1 + F3	9732	1082	1486	0.900	0.868	0.883	11,218
2nd	M1 + F4 (M2)	9725	1071	1493	0.901	0.867	0.884	11,218
3rd	M2 + F1 (M3)	9754	1062	1464	0.901	0.870	0.885	11,218
3rd	M2 + F2	9752	1080	1466	0.900	0.869	0.885	11,218
3rd	M2 + F3	9758	1094	1460	0.899	0.870	0.884	11,218
4th	M3 + F2 (M4)	9785	1069	1433	0.902	0.872	0.887	11,218
4th	M3 + F3	9765	1097	1453	0.899	0.871	0.885	11,218

*F1* the stop character feature, *F2* the current word feature, *F3* the current and context word feature, *F4* the word POS tag feature, *F5* the word associative feature

**Table 5:** The performance of RTBA for CTCF recognition

Models	TP	FP	FN	P	R	F	Total
The best CRF model (Baseline)	9785	1069	1433	0.902	0.872	0.887	11,218
RTBA with original SCCSE (R1)	10,165	743	1053	0.932	0.906	0.919	11,218

With features acquired from ICTCLAS, the P, R and F-Score were all improved comparing with the baseline model. It reveals that, in spite of lacking of Chinese word segmenter in clinical domain, the segmenter for general domain may still benefit the CTCF recognition. One reason for that is the average length of general Chinese words approximates 2 [30], but almost 93% of CTCFs contain more than 4 characters in clinical domain. So, a valid CTCF may be split to more fragments by ICTCLAS, and each POS tag aids in determining the CTCF boundary.

### Evaluation of RTBA for CTCF recognition

The model R1 represented the RTBA working with original version of SCCSE (as CTCF dictionary). The best performance CRF model (M0 + F5 + F4 + F1 + F2) was chosen for the baseline model. The RTBA with original SCCSE showed an improvement in P (from 0.902 to 0.932), R (from 0.872 to 0.906) and F-score (from 0.887 to 0.919) compared with the best CRF model on test corpus. It reveals that once the most frequently used CTCFs collected, the RTBA would have a better performance than CRF model, as shown in Table 5.

We still notice that the recall, which heavily reliant on SCCSE, is not as well as the precision. Nearly 10% valid CTCFs which should be annotated with ‘B’, ‘I’ and ‘E’ were mislabeled with tag of ‘O’ by RTBA. Generally, these false negative CTCFs might be rare but more specific and significant for differential diagnosis. The main reason for the false negative is absence of enough synonyms in Chinese clinical nomenclature. Moreover, plenty of CTCFs are still not collected in SCCSE because of various modifiers mingled within CTCFs, and different parts of a clinical findings may even be distributed in different clauses, for example, the CTCF ‘大便表面带血’ (The stools are blood-stained in the surface) is daily expressed as ‘大便干结,表面带血’ (The stools are dry, and blood-stained in the surface). This situation is not uncommon but hard to be recognized by RTBA.

Besides the false negative, the most common reason for false positive is the overlapping ambiguity, a popular phenomenon in Chinese word segmentation. For example, considering a character string ‘最高血压’, if it can be segmented into different words either as ‘最高|血压’ (the highest | blood pressure) or ‘最|高血压’ (the most | high blood pressure) depending on different context, the string is called an overlapping ambiguity string (OAS). For the reason that the SCCSE contains the CTCF ‘高血压’ (high blood pressure), the RTBA with RMM may lead wrong segmentation. That why the ‘*Exception with context’* (Table 1) was designed as a resolution. Fortunately, the clinical OASs are less popular than the general text, and most of the OASs which may lead wrong segmentation could be manually collected.

After the experiment, the new valid CTCFs in test corpus were manually added to the SCCSE again, and the word list of ‘*Exception with context’* was also refined (model R2). As a result, the F-score of RTBA reached 0.999 on test corpus. The result shows that the difficult issues of word sense disambiguation in many fields [31] might not be a big issue in dealing with refined Chinese clinical text.

### Evaluation of new CTCF prediction

**Fig. 10:**
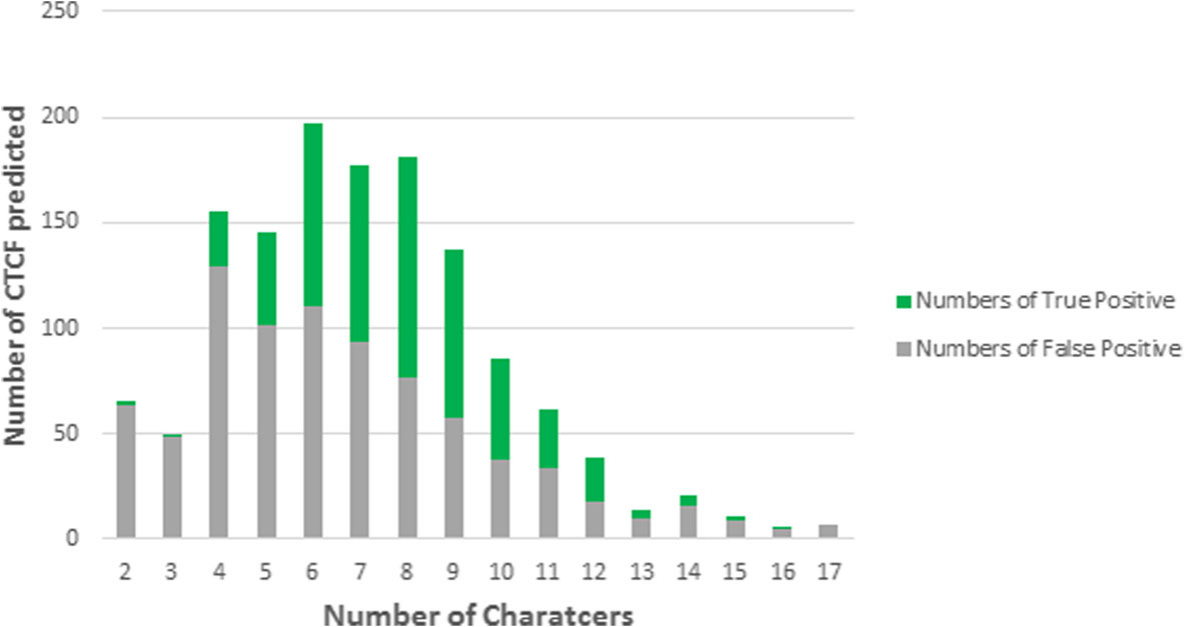
The CTCF predicted by CRF model

Though the RTBA has a better performance than CRF model in our task, it over-reliant on the over-complete nomenclature with enough synonyms. To discover new CTCFs, a hybrid method was adopted. Reviewing the test corpus, we notice that there were 535 out of 1355 phrases, which marked by CRF while unmarked by RTBA, were accord with the manual annotation. That implies, by combination of two methods, the CRF has some ability to predict new CTCFs. The character number of the marked phrases by CRF varied from 2 to 17, and arose most frequently with 4 – 9, and a high true positive performance occurred with 6 – 12 (Fig. 10). The n-gram feature UBTQ, which has a better performance in precision (true positive), helps the new CTCF selection more effective.

With manual review, the semi-automatic approach could become an iterative procedure to predict new CTCFs besides CTCF dictionary. These phrases with high frequency should be reviewed first. After several iterations, another 8,000 new CTCFs were located in the previous stage of CTCF dictionary creation.

## Discussion

A Chinese version of SCT could make up for the deficiency of Chinese EHR standards [10]. Though China is currently not the member of the International Health Terminology Standards Development Organisation (IHTSDO, who owns the SCT), the research is still a beneficial attempt for SCT localization in China. Once the membership confirmed, this human-computer interactive approach could quickly improve the daily used synonyms adding, which make the nomenclature more accessible. Beyond that, the Chinese clinical synonyms could still cross map to other international nomenclature with other available Chinese terminologies, such as MeSH. Even without any cross mapping, the CTCF dictionary itself is a standalone product which aids in Chinese term recognition without semantic web supporting.

In this research, a system which applies series of NLP techniques was proposed to retrieve CTCFs from daily physician notes in CIS, and map them to the corresponding SCT concept. The enrichment leads to the possibility of Chinese clinical information utilization in hierarchical structure of SCT. As source data for other up-layer research, a minimal deviation in text retrieving might result in wide divergence. For this reason, it’s high-risk for solving all practical problems merely depend on intelligent programs without human supervision, especially the semantic concept mapping across different language. That is why we expect that every steps of this research could be reviewed by clinical expert, even though which involves huge human workload. With human acting as a gatekeeper in CTCF validation and concept mapping, the most optimal learning approaches or parameters may not be the most crucial element.

As mentioned above, both existed rCCNs are translational version, with formal, precise and academic expression, comparing with the more complex daily expressions in real world. Surprisingly, only 4.6% descriptions were consistent in the two rCCNs, and 1.6% descriptions from the three resources (two rCCNs and the CTCF dictionary from CIS) were consistent (Fig. 2). With personal writing habit, one concept expression may vary in word sequence, abbreviation, vernacular or misspelling, and most synonyms in physician notes were still uncollected in existed rCCNs. A translated concept description may vary a lot across different translation style. This indicates that the synonym problem is widespread in Chinese clinical field, and the categorization of them has a practical significance.

There are still some obstacles to evaluate the quality of manual concept mapping, which essentially a problem of ontology enrichment. For example, some compound, multi-word CTCFs could access more than one reasonable hierarchic path, and therefore have to map to the ‘large’ or more general concepts. Some CTCFs could barely map to ‘other’ or ‘other specified’ concept categories, but these categories would never exist in daily expression. Researchers even in the same biomedical area usually evaluate different aspects of ontology enrichment, and thus cannot be compared [26]. Nowadays, most concept mapping researches are evaluated against the manual annotation, rather than ontology level. Even so, the concept mapping from experienced physicians is still practical and efficient for clinical up-layer research, such as feature selection of risk factor analysis or prognosis prediction, with CTCFs arranged in SCT semantic hierarchy. For each patient, not only the valid CTCFs were considered the features, but also their parent concepts via the IS-A relationship in SCT. Once a feature is true, its parent concept features are also true through the hierarchy. Each CTCF feature could be marked ‘positive’, ‘negative’ or ‘not-mentioned’ by scanning HPI, as a Bag-of-Concepts model instead of Bag-of-Words model [32]. Besides, the CTCFs from inpatients could be used for knowledge mining of co-morbidity relations and disorder-finding relations [33].

For literal similarity, the Chinese synonyms with a same suffix (usually serve as the *predicate*) are more similar than which with a same prefix (usually serve as the *subject*), and the literal similarity metric of AJWD, which proposed in this article, would be more suitable for Chinese. The abbreviation is also popular in Chinese, such as ‘甲旁亢’ short for ‘甲状旁腺功能亢进’ (hyperparathyroidism). It reveals that, some other literal similarity metrics, like Smith-Waterman Distance, are worthy of research in future. Besides, the misuse of the characters, such as ‘性’ and ‘型’ (both mean type), ‘症’ and ‘征’ (both mean sign),is sanctioned by usage, and either of them are accepted.

For long-term terminology maintenance, we recommend that the hospital information center introduced the low-cost crowdsourcing mechanism [34]. Physician from different department who needs text information should review or even adjust the machine-generated mapping results, in favor of SCCSE refinement. A web tool on the intranet could be developed for supporting the continuous maintenance. In several iterations, the bad mapping could be ruled out and the appropriate mapping would be determined.

Enriching a current nomenclature with another language is not just a procedure of translation, but also ontology enrichment. At present, the procedure of enrichment is largely manual, and concepts, synonyms and their relationships must be added one by one [26]. Informatics tools could accelerate this manual process. Firstly, a manual translational nomenclature is essential for semantic concept mapping across languages, even which are expensive and likely to result in low coverage. The bilingual dictionaries of medical terms from parallel corpora or other NLP technologies, such as English-Greek, English-Romanian [35] or English-Swedish [36], should be used with caution before manually reviewed by domain experts and ontologists. Secondly, since natural language in clinical text is rich and varied, a rich set of synonyms is important for NLP task. As NLP often uses ontological knowledge to interpret the texts, NLP can also help to enrich and enhance the linguistic realization of ontology [26]. The Swedish version of SCT currently suffered the same problem of synonyms lacking. Henriksson [37] used Random Indexing and Random Permutation, two distributional models, to extract synonyms from electronic health records. For lacking of enough manual annotation for Swedish evaluation, this method had to be evaluated using the English version of SCT, and 16–24% of known synonyms were identified [38]. Thus, a large-scale of manual annotation not only enrich the nomenclature, but also help for NLP methodological evaluation. Grabar [39] proposed a novel method for the automatic acquisition of French medical synonymy resources. It enables to decipher hidden synonymy relations between simple words and terms on the basis of their syntactic analysis and exploitation of their compositionality. The method has been applied to the UMLS subset of French terms and shows 99% precision. Schlegel [40] examined methods for aligning concepts in SCT with articles in Wikipedia so that newly-found synonyms could be added to SCT. They used Wikipedia redirects as a synonym source to increase the number of synonyms in SCT by 183,100 with precision of 85.6%. For language difference, most biomedical resources are not suitable for Chinese, and some NLP techniques are also limited. However, the semantic similarity approach is frequently used in Chinese synonyms enriching. Ning [41] developed a method that automatically performed ICD-10 code assignment to Chinese text diagnoses, with F-score of 91.08% on 16,330 coding instances. This process was somewhat similar to synonyms enriching, and semantic similarity estimation was applied.

For the recognition task may varied itself by the task definition and the corpus annotated, it’s hard to compare the results in different task. However, the general trends may still be comparable. In the i2b2 challenge [21], the most effective concept extraction system (used CRF) achieved an exact F-score of 0.852, which is a little lower than our 0.919 fetched by RTBA, and 0.887 fetched by CRF model. But in our research, only concepts of clinical finding were considered, which ease the burden of recognition task. Another two similar experiments, which focused on clinical finding, achieved the F-score of 0.91 [42] and 0.92 [43] respectively. The most comparable study, carried out by Wang on traditional Chinese medicine, showed a better performance (F-score of 0.951) than the results presented here [25]. One reason for the difference might be that traditional Chinese medicine record is more rigid than present days.

Although the RTBA has obtained some achievements, the diversity from multiple institutions still presents great challenge. Future work will focus on the SCCSE validating against different hospitals and attracting more users for CTCF utilization and validation. More hybrid similarity metrics which based on physician orders or remedies could be tested for CTCF categorization. Additional, the CRF could be trained on textual features enhanced with the output of a rule-based named entity recognition system [44]. The RTBA annotation would serve as not only a baseline evaluation for other learning systems, but also a training feature for other learning methods.

## Conclusions

This research shows that some NLP methods previously applied on English are also suit for Chinese clinical text. The study has demonstrated that it is feasible to help the SNOMED CT with Chinese synonyms enrichment (SCCSE) based on physician notes in CIS. With algorithms and manual validation, the CTCFs have been located, categorized, and partly mapped to the corresponding concept of SNOMED CT. As a result, totally 59,000 valid CTCFs from CIS and 11,183 SCT concept mappings have been confirmed by manual validation. Two Chinese clinical term recognition methods were proposed and the RTBA with an over-complete CTCF dictionary outperforms the CRF model. The two methods could be combined to predict new CTCF from text in CIS to enrich the SCCSE. With the continuous maintenance, the CTCFs arranged in semantic hierarchy of SCT could greatly improve the clinical up-layer research in China, especially the meaningful use of electronic health records [45]. The methodology is also useful for clinical synonyms enrichment in other languages.

## Abbreviations

AJWD: Adjust Jaro-Winkler distance
CRF: The conditional random field
CTCF: Chinese term of clinical finding
CWS: Context window size
HPI: History of present illness
JWD: Jaro-Winkler distance
POS: Part of speech
rCCN: referenced Chinese clinical nomenclature
rCTCF: referenced Chinese term of clinical finding
RTBA: The rule- and terminology-based approach
SCCSE: SNOMED CT with Chinese synonyms enrichment
SCT: SNOMED CT, short for systematized nomenclature of medicine, clinical terms
